# Functionalized Graphene Oxide with Chitosan for Dopamine Biosensing

**DOI:** 10.3390/jfb13020048

**Published:** 2022-04-27

**Authors:** Amina Omar, Ahmed M. Bayoumy, Ahmed A. Aly

**Affiliations:** 1 Physics Department, Biophysics Branch, Faculty of Science, Ain Shams University, Al Obour 11566, Cairo, Egypt; ahmedbayoumy@sci.asu.edu.eg; 2 Nanotechnology Research Center (NTRC), The British University in Egypt (BUE), Suez Desert Road, El-Sherouk City 11837, Cairo, Egypt; 3 Neuromodulatory Networks—Neuroplasticity Groups, Leibniz Institute for Neurobiology, 39118 Magdeburg, Germany; ahmed.aly@lin-magdeburg.de

**Keywords:** graphene oxide, chitosan, dopamine, molecular modeling, biosensor, QSAR

## Abstract

Detecting biological structures via a rapid and facile method has become a pronounced point of research. Dopamine (DA) detection is critical for the early diagnosis of a variety of neurological diseases/disorders. A study on the real-time optical detection of DA is described here using graphene oxide (GO) functionalized with chitosan (Cs). Hence, a computational model dependent on a high theoretical level density functional theory (DFT) using the B3LYP/LANL2DZ model is carried out to study the physical as well as electronic properties of the proposed interaction between GO functionalized with Cs and its interaction with DA. GO functionalized with a Cs biopolymer was verified as having much higher stability and reactivity. Moreover, the addition of DA to functionalized GO yields structures with the same stability and reactivity. This ensures that GO-Cs is a stable structure with a strong interaction with DA, which is energetically preferred. Molecular electrostatic potential (MESP) calculation maps indicated that the impact of an interaction between GO and Cs increases the number of electron clouds at the terminals, ensuring the great ability of this composite when interacting with DA. Hence, these calculations and experimental results support the feasibility of using GO functionalized with Cs as a DA biosensor.

## 1. Introduction

Graphene oxide (GO) has an enormous potential for use as a optical biosensor because of its distinguishing features such as the coexistence of a sp^2^/sp^3^ structure, facile surface modification, large surface area, and fluorescence capacity [1,2,3]. GO has a high reactivity, which may be attributed to having three active functional groups (hydroxyl, carboxyl, and epoxy), thus reflecting its highly reactive chemical structure and enhancing its potentiality for various biological applications [4].

Chitosan (Cs) is a biopolymer derived from chitin. It is a non-toxic, biocompatible, bioactive, and biodegradable polymer. Cs has a good film-forming ability, which allows it to be widely used for the development of biosensors. Cs can be used as an immobilization platform for biomolecules in electrochemical and optical biosensors [5,6].

Dopamine (DA) is a major catecholamine neurotransmitter playing essential roles in the cardiovascular and central nervous systems. As such, high DA levels lead to cardiotoxicity, further leading to rapid heart rates, hypertension, and heart failure [7]. On the contrary, low levels of DA in the central nervous system are implicated as a major cause of several neurological diseases such as Parkinson’s disease, schizophrenia, and Alzheimer’s disease [8]. Therefore, it is obvious that DA measurements are required to understand its biological functions and related biological processes and mechanisms. There were several techniques used for measuring DA, but sometimes, the costs were high. Therefore, recently, researchers have been interested in developing a low-cost biosensor surface for DA detection. There are several ways to sense DA, with optical sensing using nanostructured materials such as GO providing many advantages such as a rapid and simple assay design [2,9,10]

On the other hand, the concepts and tools of molecular modeling are applied to assess the nature of interactions among a proposed sensor’s components on a theoretical basis. The principal aim of constructing a molecular model is enhancing and explaining the result of the experimental work on a mathematical basis to study the physical as well as biological features of chemical structures via computer soft codes, which can frequently present crucial data in several interdisciplinary branches of science [11,12,13]. Not only do theoretical models play vital roles in cases where experimental work is hampered, or for ethical or situational reasons, but also they are able to offer reasonable elucidations for current work based on theoretical concepts [14,15]. Quantitative structure activity relationship (QSAR) calculations always provide a potential route for studying structures due to its simple strategy for forecasting their biological activity. Such a methodology can present the quantitative relationship between the physicochemical properties of biological structures and their biological activities [16]. Its ability to determine some QSAR parameters in terms of various mathematical equations that directly and/or indirectly illustrate the assessed bioactivities of biological molecules under investigation have been proven [17,18]. Therefore, calculating the QSAR descriptors has become one of the rising trends within the scientific community [19,20,21,22,23]. Moreover, constructing a mapping electrostatic potential (MESP) has boosted the pronounced uses of molecular modeling computations [24]. They are usually created for their significant ability to highlight the reactive sites of studied chemical compounds. They are characterized by being quite accurate in describing these active sites [25]. Moreover, they are promising in evaluating the types of chemical addition reactions: nucleophilic or electrophilic. Molecular surfaces were previously considered to be as presented as in References [26,27,28,29,30,31]. However, the MESP helped to visualize the charge distributions of molecules, so it has become invaluable in predicting the behavior of biological molecules.

In this work, the GO functionalized with Cs and its interaction with DA are studied theoretically at the DFT level using B3LYP/LAN2DZ, and its physical and biological properties are studied based on calculations of the QSAR and MESP. Then, a GO-based membrane biosensor is developed for real-time optical detection of DA.

## 2. Calculation Details

All energy calculations for the proposed structures were conducted via Gaussian 09 software [32] at the Spectroscopy Department, National Research Centre (NRC), Egypt. The calculations were carried out using DFT theory at the B3LYP level with the LANL2DZ basis set [33,34,35]. Some physical parameters were extracted from the calculated structures such as total energy (E), total dipole moment (TDM), and HOMO/LUMO bandgap energy (ΔE). Then, MESP maps were constructed at the same theoretical level. After that, the suggested structures were geometrically optimized at the semiemprical quantum mechanical calculation level via the PM6 method [36] utilizing SCIGRESS 3.0 soft code, which was executed at the Spectroscopy Department, Physics Division, National Research Centre, NRC [37]. The QSAR parameters, such as, Log P, heat of formation (HF), ionization potential (IP), molar refractivity (MR) and polarizability (P), were calculated after the optimization processes and descriptors.

## 3. Materials and Methods

### 3.1. Materials

The Cs (molecular weight 100,000–300,000 from ACROS), graphite, sulfuric acid (H_2_SO_4_), orthophosphoric acid (H_3_PO_4_), potassium permanganate (KMnO_4_), hydrochloric acid (HCl), and hydrogen peroxide H_2_O_2_ (30 *v*/*v* %) used in this study were obtained from Loba Chemie. DA hydrochloride was purchased from Sigma. A PBS buffer solution (pH 7.0) was used in the experiments.

### 3.2. Synthesis of Graphene Oxide (GO)

The GO was prepared according to a modified Hummers’ method [38]. H_2_SO_4_ and H_3_PO_4_ were mixed at a volume ratio of 9:1 (180:20 mL) and stirred for 15 min. Then, 1.5 g of graphite powder was added into the mixture before 9 g of potassium permanganate (KMnO_4_) was gradually added while the mixture was stirred. The reaction was stirred for 48 h at room temperature. After the 48 h, 4 mL of H_2_O_2_ was poured into the mixture to stop the oxidation reaction. The mixture was then separated into centrifuge tubes for cleaning. The cleaning process was performed using HCl and deionized water centrifuged at 4000 rpm for 10 min. This process was repeated twice; then, the product was dried in an oven at 80 °C for 24 h.

### 3.3. Preparation of Cs/GO Composite Membranes

Different concentrations (25 mg, 50 mg, and 75 mg) of the prepared GO was dispersed into 50 mL of a 0.5% Cs solution dissolved in 1% (*v*/*v*) acetic acid to form homogenous mixtures of Cs/10% GO, Cs/20% GO, and Cs/30% GO, respectively, followed by stirring for 1 h. After that, the homogeneous GO/Cs solutions were poured into glass plates and kept at 100 °C for 4 h.

### 3.4. Fluorescence Measurements of GO and Cs/GO Composite Membranes with DA

The prepared Cs/GO membranes with different ratios of GO (Cs/10% GO, Cs/20% GO, and Cs/30% GO) were used for DA detection. A 50 µM DA in pH 7 PBS was selected as a medium concentration of DA, which was the concentration used in previous research [39]. These samples were put into 50 µM DA in pH 7 PBS, mixed thoroughly, and incubated in water bath at 40 °C for 10 min. Then, the solutions were taken out for the fluorescence measurements at an excitation wavelength of 450 nm and at an emission wavelength of 530 nm.

### 3.5. Real-Time Fluorescence Detection of DA on Cs/GO Composite Membranes

The Cs/GO sample was used to detect DA over time; 50 µM DA in pH 7 PBS solution was dropped onto the prepared GO/Cs membrane. Then, the fluorescence intensity was measured over time from 1 s to 3 min.

### 3.6. Characterization Techniques

All prepared samples were characterized using spectroscopic techniques using an Attenuated Total Reflection Fourier Transform Infrared (ATR-FTIR) spectrometer (Vertex 80, Bruker) with spectral range 4000–400 cm^−1^. Additionally, the prepared GO structure was investigated based on X-ray Diffraction (XRD) measurement using a Malvern Panalytical Empyrean 3 diffractometer. Finally, all fluorescence measurements were performed on a Cary Eclipse fluorescence spectrophotometer and inverted fluorescence microscope (Zeiss Axio Observer 5).

## 4. Results and Discussion

### 4.1. Building Model Molecules

**Figure 1:**
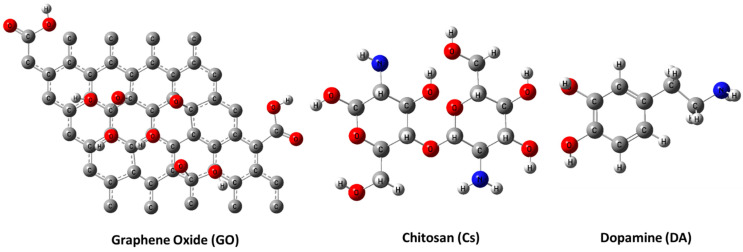
Molecular models of GO, Cs, and DA calculated using DFT:B3LYP/LANL2DZ(C in grey, H in white grey, O in red, and N in blue).

**Figure 2:**
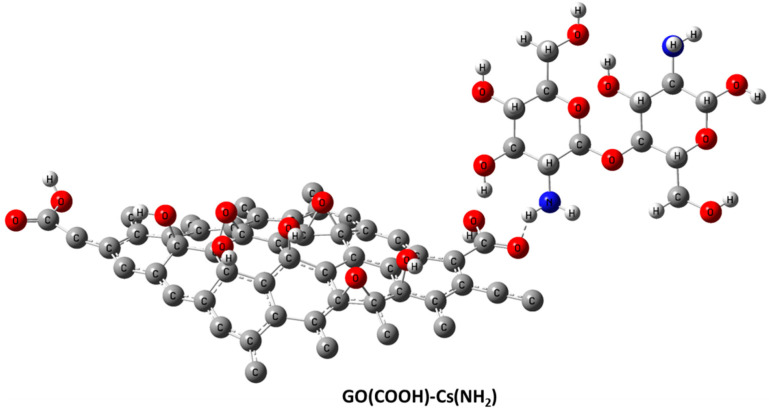
Molecular model of Cs absorbed at the terminal of GO calculated using DFT:B3LYP/LANL2DZ (C in grey, H in white grey, O in red, and N in blue).

**Figure 3:**
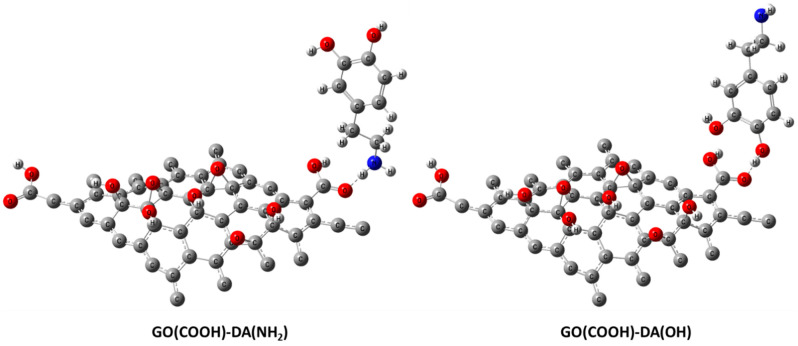
Molecular models of GO(COOH)-DA(NH_2_) and GO(COOH)-DA(OH) calculated using DFT:B3LYP/LANL2DZ (C in grey, H in white grey, O in red, and N in blue).

**Figure 4:**
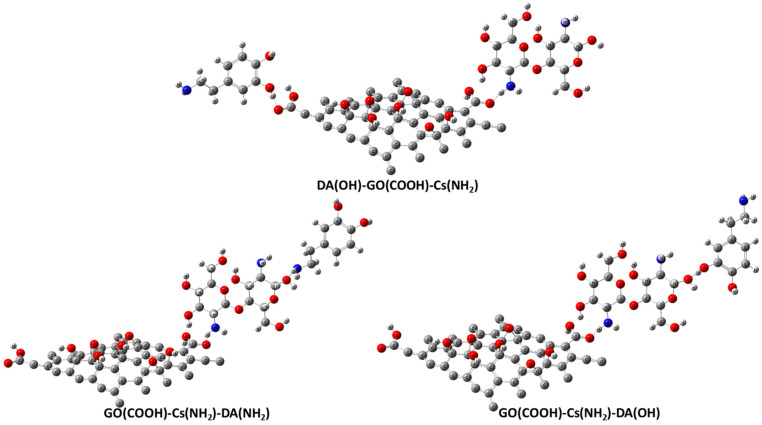
Molecular models of DA(OH)-GO(COOH)-Cs(NH_2_), GO(COOH)-Cs(NH_2_)-DA(NH_2_), and GO(COOH)-Cs(NH_2_)-DA(OH) calculated using DFT:B3LYP/LANL2DZ (C in grey, H in white grey, O in red, and N in blue).

Figure 1 depicts the molecular models of the proposed individual structures: GO, Cs, and DA. It is worth noting that these models are constructed based on previous work by our research group; for example, the GO structure is constructed based upon the previously published work of [40], while both Cs and DA were presented in [41]. In addition, GO is the main component of the proposed sensor; hence, the interaction between pristine GO and our target biomolecule DA is proposed, and GO functionalized with Cs biopolymers interacting with DA is investigated too. Since DA has two functional groups (OH and NH_2_), they are anticipated to form an H bond with the most reactive site of GO (the COOH group). There are several possibilities for this GO and Cs interaction, but the interaction between O of the carboxyl COOH group of GO with NH_2_ of Cs through a physical interaction (H bonding) is selected based upon the conclusion of the work in [40] (Figure 2). Figure 3 illustrates the two possible interactions between GO and DA. In the same manner, Figure 4 shows the interaction probabilities of GO-Cs interacting with DA; one possibility is on the GO side, and the two others lean toward the Cs NH_2_ functional group.

### 4.2. Energy Calculations

Calculations of the total energy were conducted for the molecular models constructed including GO, Cs, and DA and for their various interaction probabilities through hydrogen bond formation at a high theoretical level (DFT) using the B3LYP/LANL2DZ model. The calculated physical and electronic parameters such as total energy (E), TDM, and HOMO/LUMO bandgap energy (ΔE) are listed in Table 1.

Table 1 lists some of the calculated parameters such as energy, TDM, and HOMO/LUMO bandgap energy that reflect both the physical and electronic characteristics of the chemical structure under investigation. The total energy as one of the physical indicators of the structure’s stability equals −75.3873, −34.2231, and −14.0558 keV for GO, Cs, and DA, respectively. GO functionalization with Cs results in a blend with a much lower energy (−109.6286 keV) and hence a much higher stability. The interaction between DA and GO, either through the OH or NH_2_ groups, produces two structures with roughly the same energies (~−89 keV) indicating the same level of stability. In the same manner, the addition of DA to functionalized GO yields structures with the same energy and stability. This ensures that GO/Cs blends are stable structures forming a highly firm interaction with DA, which are energetically preferred. TDM is considered one of the physical indicators of a structure’s reactivity. It is calculated to be 14.1457, 5.4576, and 2.7755 Debye for GO, Cs, and DA, respectively. The high reactivity of GO may be attributed to having three active functional groups (hydroxyl, carboxyl, and epoxy). This reflects a highly reactive chemical structure, which enhances its potentiality for various applications. However, the interaction between DA and GO lowers GO’s dipole moment from 14.1457 to 8.887 Debye for the NH_2_ group and to 11.746 Debye for the OH group, thus lowering its reactivity. Contrary to the energy calculations, DA with its OH group yields more reactive structures than with its NH_2_ group. This reflects a preference for the OH group of DA. A dramatic rise in the calculated TDM of GO functionalized with Cs occurs (35.1547 Debye), reflecting an extremely active structure. Opposed to the GO-DA probability, the interaction of DA with functionalized GO produces structures with significantly large TDMs: 35.9173, 28.9391, and 32.5384 Debye for DA(OH)-GO(COOH)-Cs(NH_2_), GO(COOH)-Cs(NH_2_)-DA(NH_2_), and GO(COOH)-Cs(NH_2_)-DA(OH), respectively. This reflects an extreme reactivity and ensures the impact of the OH group of DA on preserving the reactivity of GO/Cs blend. Finally, the HOMO/LUMO bandgap energies are considered as reflecting the electrical conductivity of the suggested structures. ΔE is 0.0022 eV for GO, while it equals 0.1045 and 0.156 eV for Cs and DA, respectively. A sharp decline occurs for the calculated ΔE for DA interacting with GO through its NH_2_ group while a slight drop is noticed for the interaction through its OH group. The addition of Cs to GO halved its calculated ΔE and doubled its conductivity. However, the interaction of DA with functionalized GO elevates the resulting ΔE to a maximum value of 0.0058 eV for the DA(OH)-GO(COOH)-Cs(NH_2_) structure. Therefore, the calculated physical and electronic parameters ensure the feasibility of functionalizing GO with Cs and their ability to interact with DA biomolecule, yielding a highly stable and extremely reactive structure.

### 4.3. Molecular Electrostatic Potential (MESP) Maps

**Figure 5:**
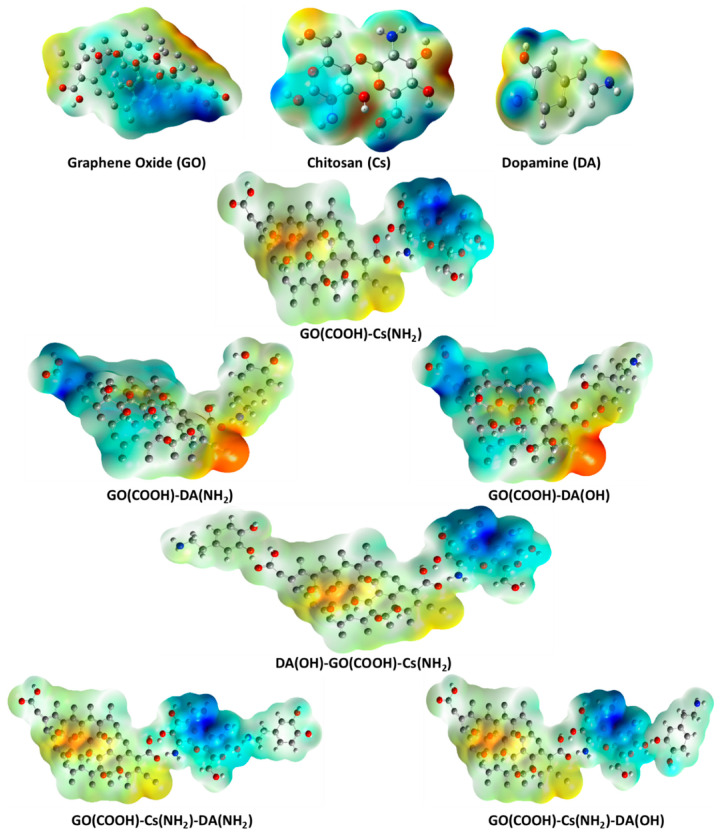
Calculated MESP maps for GO, Cs, DA, GO(COOH)-Cs(NH_2_), GO(COOH)-DA(NH_2_), GO(COOH)-DA(OH), DA(OH)-GO(COOH)-Cs(NH_2_), GO(COOH)-Cs(NH_2_)-DA(NH_2_), and GO(COOH)-Cs(NH_2_)-DA(OH) calculated at DFT level using the B3LYP/LANL2DZ model.

Calculating MESP maps for the suggested structures is usually attributed to their unique capabilities for visualizing the electrostatic charge distribution across a chemical structure and, hence, its chemical active sites [42]. Consequently, they are conducted for GO, Cs, and DA and their possible interactions using the B3LYP/LANL2DZ theoretical model. Figure 5 depicts all of the calculated MESP maps for the revealed structures. A typical MESP construction includes a wide range of colors, which are explained in detail in [43,44]. They bear a striking resemblance to the well-known rainbow extending from red to dark blue, differentiating between sites of high and low electronegativity. The full spectrum of MESP maps consists of red, orange, yellow, green, light blue, and dark blue. Moreover, it is well-known that binding atoms of similar electronegativity values leads to the creation of maps with light blue and yellow colors at all structural sites. This is explained by the adverse impact of each atom on the others, mutually cancelling their power.

The MESP map of GO consists of four main regions, where each one has its electric charge creating a structure with a different active site. An electronegative region appears in red on the right side of the structure and is opposite to a yellow color region of lower electric charge. These two regions most likely undergo nucleophilic reactions. On the other hand, the lower side is characterized by a dark blue region, indicting a positively charged active site. Likewise, the rest of the GO structure, which seems to have less positive charges than at the dark blue site, show regions tending to favor electrophilic interactions. The chitosan dimer structure has two distinct charges; negative charges appear obviously near the electronegative O atoms, pointing to a preference for nucleophilic reactions, and less electronegative sites (in yellow, and light and dark blue) appear around the amine groups and cyclic rings. Similarly, the DA structure shows both negatively charged sites (near the N and O species) and positive ones (near H, C, and its ring). This suggests a tendency to participate in both types of reactions. Functionalizing GO with the Cs biopolymer creates an electric charge distribution gradient from a highly positive region around the Cs structure (appearing in dark and light blue) to a quite negative sites on the GO side (orange and yellow). This refers to the great ability of GO to withdraw electrons from Cs, which may be explained by GO having high electron mobility contrary to the organic Cs structure. The MESP maps of the two suggested that the interactions between GO and DA have nearly the same configuration, meaning that no significant impact is seen for the DA interaction site. The calculated map seems to be highly electronegative in the vicinity of the proposed interaction site, which contains two electronegative functional groups (NH_2_ and COOH), suggesting that the high electron mobility region strengthens the probability of the proposed interaction. However, the negativity becomes less as we move away from the interaction site, reaching a dark blue region at the GO terminal. Likewise, interacting DA with functionalized GO results in structures with similar charge distributions regardless of the interaction site. Cs appears in dark blue, indicating a region with a highly positive charge with respect to the light blue site around DA. While GO has orange and yellow sites to show their electronegativity. This again refers to the ability of GO to attract electrons from structures when interacting with a region of high electron mobility.

### 4.4. Quantitative Structure Activity Relationship (QSAR) Calculations

QSAR descriptors are usually considered one of the unique facilities of molecular modeling simulations in studying chemical entities that have possible biological potentials. In fact, they can offer a quite facile and accessible route for forecasting their probable reactivity in biological media. Hence, it continues to be the main research point in many recent papers concerning biological activity [45,46,47,48,49,50,51]. Hence, these parameters are calculated for the investigated structures at the PM6 theoretical level. Table 2 demonstrates the calculated descriptors: Log P, heat of formation (HF), ionization potential (IP), molar refractivity (MR), and polarizability (P). According to Table 2, the proposed interaction between GO and Cs (GO(COOH)-Cs(NH_2_)) had a more negative value for Log P, which indicates the greater hydrophilicity compared with the other interaction between GO and DA. Additionally, higher IP and P indicate more reactivity for this structure when acting as a composite substrate for DA biosensing. Then, adding DA in three different positions on GO(COOH)-Cs(NH_2_), DA(OH)-GO(COOH)-Cs(NH_2_) shows high biological activity for this structure.

### 4.5. FTIR and XRD Analysis

**Figure 6:**
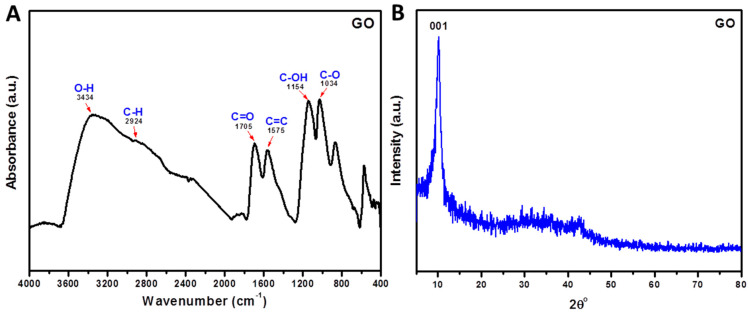
ATR−FTIR (**A**) and XRD (**B**) of prepared graphene oxide (GO).

**Figure 7:**
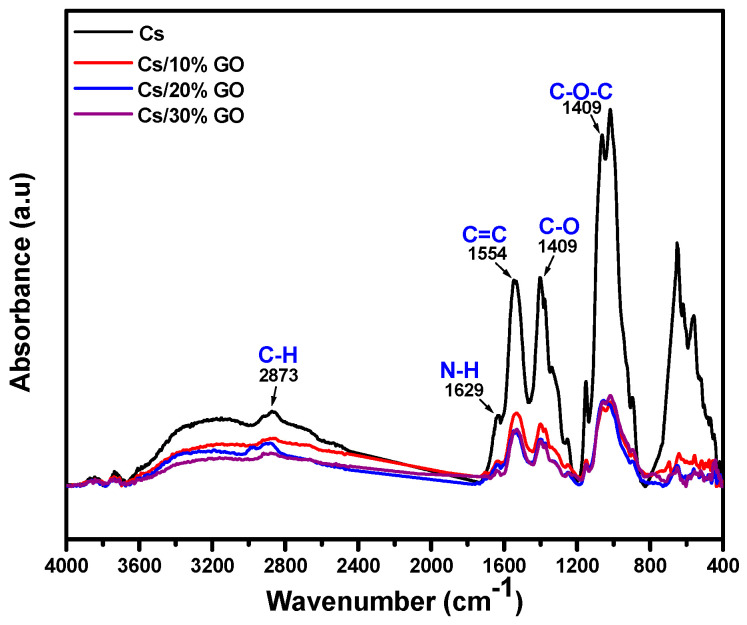
ATR−FTIR of Cs and Cs/GO composites membranes with different ratios of GO.

The ATR−FTIR spectra of GO are presented in Figure 6A. The spectra show the presence of oxygen-containing functional groups in GO. The peak at 3434 cm^−1^ shows the stretching of the hydroxyl group, C=O carbonyl stretching at 1705 cm^−1^, and the C−O epoxide group stretching at 1154 and 1034 cm^−1^. The high intensity of the major peaks in GO reveals that a large amount of oxygen-containing groups are present after oxidation. The peak at 1575 cm^−1^ is related to the skeletal stretching of the C=C alkene group. Figure 6B illustrates the XRD patterns used to identify the composition and structure of the nanoparticles synthesized. The GO diffraction peak is illustrated at about 2θ = 10°, corresponding to the (001) reflection plan [52]. Figure 7 showed the ATR−FTIR of Cs, Cs/10% GO, Cs/20% GO, and Cs/30%. The characteristic absorption bands for Cs appear in Figure 7. The amide group of chitosan appeared 1629 cm^−1^. This peak decreases in intensity when adding GO and shifts to 1642 cm^−1^. These data confirm the interaction of chitosan with different ratios of graphene oxide. These prepared membranes were ready to be used as a biosensor substrate for DA.

### 4.6. Fluorescence Measurements of GO and Cs/GO Composite Membranes with DA

**Figure 8:**
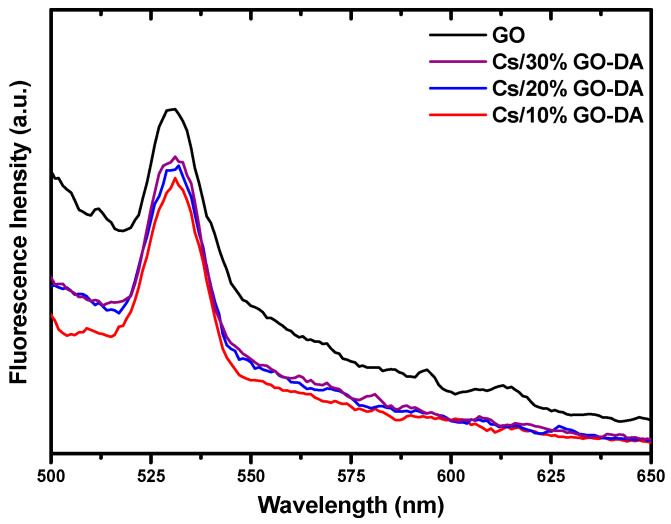
Fluorescence spectra of the GO aqueous solution and Cs/30% GO, Cs/20% GO, and Cs/10% GO with 50 µM DA in pH 7 of PBS at an excitation wavelength of 450 nm.

A strong fluorescence emission centered at 530 nm under excitation using 450 nm light appears in Figure 8. The fluorescence band of functionalized GO comes from specific electronic transitions between the antibonding and the bonding molecular orbitals, such as σ*→n, π*→n, and π*→π [53]. GO generally contains various oxygen-containing functional groups, such as hydroxyl groups (C−OH), carboxyl groups (COOH), carbonyl (C−C=O), epoxy (C−O−C), and aromatic rings. As a result, multiple fluorescence peaks, which correspond to different electronic transitions, are usually excited simultaneously. When these fluorescence peaks overlap with each other, a single broad peak is displayed. The addition of DA to different ratios of GO functionalized with Cs leads to a significant quenching of the fluorescence of GO (Figure 8). Moreover, DA gave a steady and maximal quenching effect because of the high affinity between anionic GO and positively charged DA molecules. Additionally, decreasing the ratio of GO in a prepared membrane decreases the intensity of the fluorescence, which proved a fluorescence effect of GO.

### 4.7. Real-Time Fluorescence Detection of DA on Cs/GO Composite Membrane

**Figure 9:**
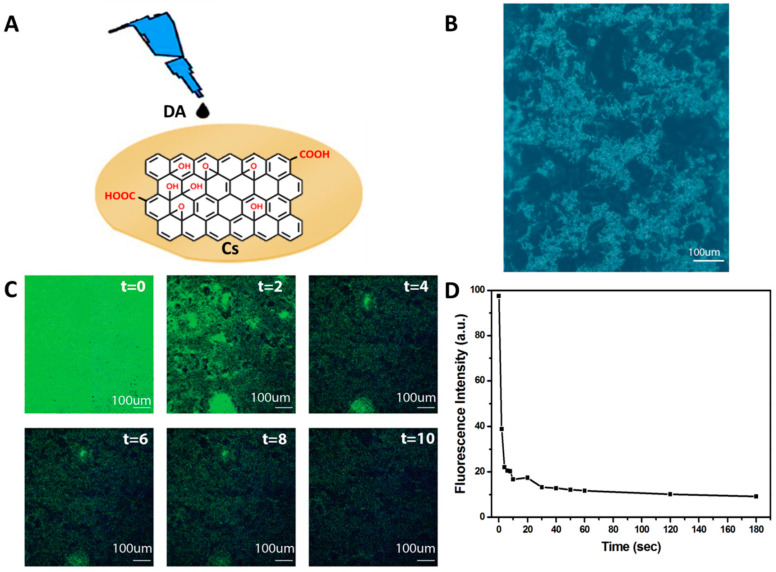
(**A**) Schematic diagram depicting the biosensing detection of DA on the Cs/GO membrane. (**B**) Bright field images of the Cs/GO membrane. (**C**) Real-time fluorescence images of the Cs/GO membrane after adding DA for 0, 2, 4, 6, 8, and 10 s. (**D**) Time-dependent fluorescence changes in the Cs/GO membrane with DA (50 µM DA in pH 7 of PBS).

A schematic diagram depicting the biosensing detection of DA on a Cs/GO membrane subsrate is shown in Figure 9A. The bright field of the Cs/GO membrane shows the microstructure of the distrubution of GO on the Cs membrane in Figure 9B. Real-time fluorescence images of the Cs/GO membrane after the addition of DA is illustrated in Figure 9C, showing the quenching of the fluorescence with the addition of DA at 0, 2, 4, 6, 8, and 10 s, and time-dependent fluorescence changes in the quenched Cs/GO membrane are illustrated in Figure 9D. The results indicate that about 90% of the fluorescence intensity is quenched by adding DA to the Cs/GO membrane substrate for 3 min, which proved the senstivity and reactivity of the GO functionalized with Cs when used as a DA biosensor.

## 5. Conclusions

GO and Cs/GO composite membranes were synthesized to be used as a biosensor substrate for DA. A theoretical approach using DFT calculations showed that GO has a high reactivity, which may be attributed to having three active functional groups (hydroxyl, carboxyl, and epoxy), and thus reflecting a highly reactive chemical structure and enhancing its potentiality for various biological applications. DFT calculations illustrate that the calculated physical and electronic parameters ensure the feasibility functionalizing GO with Cs and their ability to interact with DA biomolecules, yielding a highly stable and extremely reactive structure. The experimental approach used FTIR and XRD analyses for the prepared GO and Cs/GO composite membrane. The results of the experimental FTIR spectra and XRD support the synthesis of GO and Cs/Go composites. Real-time fluorescence-based images for the detection of DA on Cs/GO substrate are tested. By introducing DA to the Cs/GO membrane substrate for 3 min, nearly 90% of the fluorescence intensity is quenched, which ensures the sensitivity and reactivity of the GO functionalized with Cs when used as a DA biosensor.

## Figures and Tables

**Table 1:** Calculated total energy (E) in keV, total dipole moment (TDM) in Debye, and HOMO/LUMO bandgap energy (ΔE) in eV for GO, Cs, and DA and their interaction possibilities using DFT:B3LYP/LANL2DZ theoretical model.

Structure	E (keV)	TDM	ΔE (eV)
GO	−75.3873	14.1457	0.0022
Cs	−34.2231	5.4576	0.1045
DA	−14.0558	2.7755	0.1560
GO(COOH)-Cs(NH_2_)	−109.6286	35.1547	0.0011
GO(COOH)-DA(NH_2_)	−89.4631	8.8870	0.0004
GO(COOH)-DA(OH)	−89.4642	11.7460	0.0021
DA(OH)-GO(COOH)-Cs(NH_2_)	−123.6843	35.9173	0.0058
GO(COOH)-Cs(NH_2_)-DA(NH_2_)	−123.6832	28.9391	0.0050
GO(COOH)-Cs(NH_2_)-DA(OH)	−123.6839	32.5384	0.0052

**Table 2:** QSAR parameters including Log P, heat of formation (HF) in kcal/mol, ionization potential (IP) in eV, molar refractivity (MR), and polarizability (P) in A^3^ for GO, Cs, and DA and their interaction possibilities calculated at the PM6 theoretical level.

Structure	Log P	HF (kcal/mol)	IP (eV)	MR	P (A^3^)
GO	−11.799	865.987	−9.199	200.287	110.455
Cs	−3.672	−401.666	−9.827	71.652	20.257
DA	0.847	−67.794	−8.657	42.674	12.008
GO(COOH)-Cs(NH_2_)	−15.335	479.760	−8.433	277.771	128.723
GO(COOH)-DA(NH_2_)	−9.801	791.403	−8.452	244.881	116.787
GO(COOH)-DA(OH)	−10.816	819.149	−8.529	248.793	119.778
DA(OH)-GO(COOH)-Cs(NH_2_)	−12.974	393.007	−8.752	316.428	145.612
GO(COOH)-Cs(NH_2_)-DA(NH_2_)	−14.488	401.997	−8.332	320.445	141.113
GO(COOH)-Cs(NH_2_)-DA(OH)	−14.488	405.563	−8.387	320.445	141.711

## Data Availability

All original measurements and data analysis of this work will be available when required.
